# Fabrication of graphene film composite electrochemical biosensor as a pre-screening algal toxin detection tool in the event of water contamination

**DOI:** 10.1038/s41598-018-28959-w

**Published:** 2018-07-16

**Authors:** Wei Zhang, Baoping Jia, Hiroaki Furumai

**Affiliations:** 1 Research Centre for Water Environment Technology, Department of Urban Engineering, The University of Tokyo, Tokyo, 113-0033 Japan; 2 School of Materials Science and Engineering, Changzhou University, Changzhou, Jiangsu, 213164 China; 3 College of Engineering, Swansea University, Bay Campus, Swansea, SA1 8EN UK; 4 School of Natural and Built Environments, University of South Australia, Mawson Lakes, South Australia 5095 Australia

## Abstract

In this work, we fabricated a novel graphene film composite biosensor for microcystin-LR detection as an alternative to time-consuming, expensive, non-portable and often skills-demanding conventional methods of analysis involved in water quality monitoring and assessment. Excellent linear correlation (*R*^2^ = 0.99) of the electron-transfer resistance was achieved over a wide range of microcystin-LR (MC-LR) concentration, i.e. 0.005–10 μg/L. As-prepared graphene film composite biosensors can specifically detect MC-LR with remarkable sensitivity and detection limit (2.3 ng/L) much lower than the World Health Organization (WHO) provisional guideline limit of microcystin-LR concentration (i.e. 1 μg/L) in different water sources. Their great potential can be attributed to large active surface area of graphene film and efficient charge transfer process enabled by their high conductivity. Developed graphene film composite biosensors were also successfully applied to determination of MC-LR in several environmental water samples with high detection recovery, which offers a promising possibility of large-scale manufacture of sensor tips due to their macroscopic free-standing nature, the scalable fabrication route and easily tunable size.

## Introduction

Access to safe drinking water and their sufficient treatment are of paramount importance to people’s health and quality of life in any community around the world^1,2^. Ever increasing episodes of harmful algal blooms (HAB) resulted from intensified anthropogenic activities such as agricultural run-off, urban waste discharge, and manufacturing of detergents has exerted a significant strain in drinking water access worldwide, especially where drinking water treatment plants receive from surface water sources. Since they contain harmful cyanobacteria, which are also known as blue-green algae and can produce and release highly potent cyanotoxins to the surrounding environment, their occurrence has become serious hazards to local public health. Estimated 50 to 70% of intracellular metabolite released during a cyanobacterial HAB are harmful toxins (i.e. cyanotoxins)^3,4^, such as hepatotoxins (microcystins, cylindrospermopsin, and nodularin), neurotoxins (brevetoxins, anatoxins and saxitoxins), and dermatoxins (lyngbyatoxins, lipopolysaccharides)^5–8^. Microcystins is one of most frequently detected cyanotoxin throughout the world and some variants of microcystins have been claimed to be bioaccumulative through the biological chain. To date, 80 variants of microcystins have been isolated and identified from freshwater cyanobacteria genera, among which microcystin-LR (MC-LR) is the most toxic variant^9–11^, with a LD_50_ value of 43 μg/kg for mouse bioassay 12^12^. Due to its severe toxicity, the provisional guideline concentration limit of 1 μg/L MC-LR in drinking water was assigned by the World Health Organization (WHO) in 1998^13^. Following a significant HAB event, there is an urgent need to establish when a water source is safe to use or to evaluate the level of treatment required to make a source safe using a cost-effective generic analysis platform. These platforms should be easily tailored to provide early warnings of contamination episodes and allow reliable screening in water quality from catchment to consumer. Well-established methods to detect MC-LR in water include high-performance liquid chromatography/mass spectrometry (LC/MS)^14^, bioassays^15^, biochemical assays^16^, and immunoassays^17^, which often require long processing times, sophisticated instruments, complex procedures, or high processing cost and are in general used in the laboratory, not *in situ*.

In the last decade, there have been significant progresses in developing sensitive and specific electrochemical biosensors/immunosensors, which make suitable devices for *in situ* monitoring MC-LR due to their possible miniaturisation, portability and automation^18,19^. The unique structure of single carbon atom layers arranged in a hexagonal lattice give graphene sheet superior physical and electrochemical properties (e.g. high electrical conductivity, ease of functionalization, high electrochemically active surface area, and broad range of working potentials in aqueous solutions)^20–24^. Consequently, many efforts have been made to utilize these outstanding properties of graphene for macroscopic applications such as flexible/stretchable electronics as alternatives to the time-consuming, expensive, non-portable and often skills-demanding conventional methods of analysis involved in water quality assessment^25–28^. However, their fabrication in terms of desirable form and better morphological control is still challenging at the moment. For example, mechanical exfoliation of graphite can produce high quality single graphene sheets, but it is laborious with a low yield, and is unlikely to be a good route for commercially viable devices. Hence, special emphasis has been placed to find an economic and scalable fabrication route which will allow graphene to be exploited more fully in this direction. Recently, modified chemical vapour deposition (CVD) method to grow large-sized monolithic graphene/polymer sheets featuring multilayer graphene deposition has been developed (i.e. the roll-to-troll method)^29^. Firstly, carbon-containing gaseous species (i.e. methane) react at high temperatures (900–1100 °C) in the presence of a metal catalyst, which serves both in the decomposition of the carbon species and in the nucleation of the graphene lattice^30^. Using copper as catalyst substrate can lead to the surface deposition of very thin graphene layer due to its very low carbon solubility (i.e. 0.001 atomic %). In addition, thin foil of copper has the advantage of great flexibility and thus highly compatible with the roll-to-troll method^31,32^. Following the CVD process, grown graphene film (GF) are ready to be transferred to flat or curved polymeric support on demand, which reportedly consisted of three essential steps: (1) adhesion of polymer supports to the graphene on a Cu foil, (2) etching of Cu layers removal by electrochemical reaction with a Cu etchant, and (3) release of graphene layers and transfer on to a target substrate by removing the adhesive force on the polymer support. In the adhesion step, the GF grown on a Cu foil is attached to a thin polymer support such as thermal-release tapes between two rollers. The whole transfer process can be repeated to produce stacks of graphene layers onto the polymer substrates. The scalability and the processibility of CVD and roll transfer methods provide one step further towards developing practical graphene-based biosensors in large scale as the resulting graphene material is free standing, scalable and macroscopic with easily tuneable size rather than previously reported two dimensional nanoflakes or their agglomerates often in fine powder form.

In this work, we fabricated a GF electrochemical sensor for MC-LR detection in water, which essentially consisted of three steps. Firstly, GF were synthesized first on Cu foil using a modified CVD method and then transferred onto polyethylene terephthalate (PET) substrate via thermal release method. The surfaces of GF/PET electrodes were then functionalized by electrochemical oxidation in an alkaline solution to increase oxygen containing functional groups (i.e. carboxyl group). Secondly, GF biosensors were prepared by conjugating MC-LR via biomolecule cross-linking agents to the functionalized GF electrode surface. Incubation solutions containing monoclonal antibodies specific to MC-LR were prepared, which can provide the required specificity to detect the cyanotoxin. The electron-transfer resistance changes of the functionalized graphene electrodes after the MC-LR conjugation and after exposure to antibodies in the incubation solutions were then used to measure the variation of MC-LR concentration and assess the GF sensing performance. Consequently, a linear response curve of the electron-transfer resistance versus the MC-LR concentration in the range of 0.005–10 μg/L were achieved with a detection limit well below that of WHO provisional MC-LR concentration in drinking water (i.e. 1 μg/L). Finally, much improved detection range of MC-LR (i.e. an order of magnitude lower) and biosensor linear response (i.e. *R*^2^ > 0.99) were achieved in comparison with our previous study using another type of graphene electrode, which was probably due to more uniform coating of graphene film^33^.

## Materials and Methods

### Fabrication of GF biosensor electrodes

**Figure 1:**
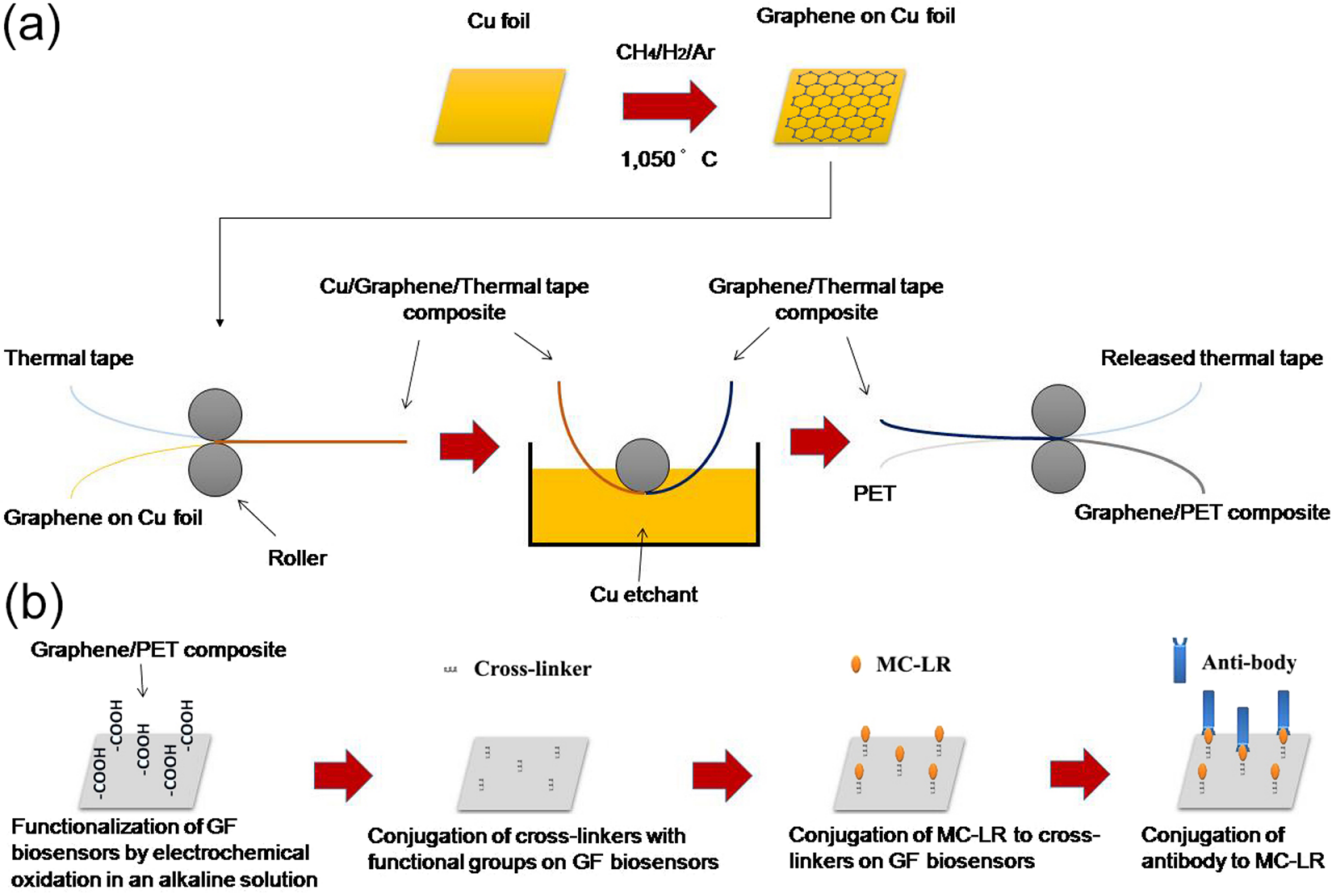
Schematic illustrations of GF biosensor electrode synthesis procedure (**a**) and preparation procedure (**b**) for MC-LR detection in water.

Figure 1(a) illustrates a simplified GF biosensor electrode synthesis procedure. Cu foils (25 μm thick, 99.8%, Alfa) as graphene growth substrate was cut into pieces of 10 × 10 mm^2^ and cleaned by acetone, methanol, and deionized water. During CVD process, Cu foils were first heated up to 950 °C in a horizontal quartz tube furnace under Ar (100 s.c.c.m.) and H_2_ (200 s.c.c.m.) and annealed for 15 min to clean their surfaces and increase the Cu grain size. Graphene growth was then carried out at 1050 °C by introducing CH_4_ of different concentrations balanced in Ar and H_2_ with a total flow rate of 1500 sccm (1.3% H_2_) for growth time between 5 and 60 min. After growth, samples were rapidly cooled to room temperature at a rate of ~100 °C/min in the protection of Ar (500 s.c.c.m) and H_2_ (200 s.c.c.m.).

In next step, GF grown on the Cu foil was attached to a thermal release tape (Nitto Denko Co.) by applying soft pressure (~0.2 MPa) between two rollers. After etching the Cu foil in a plastic bath filled with etching solution of iron nitrate (0.05 g/mL) for around 12 h, the transferred GF on the tape was rinsed with deionized water to remove residual etchant. Subsequently, the GF on the thermal release tape was inserted to the rolls together with 130 µm thick polyethylene terephthalate (PET) substrates and exposed to mild heat of 90~120 °C for 3~5 min, resulting in the transfer of GF from the tape to the target substrate.

### Characterization of GF electrochemical sensor

For charactering the morphology of GF electrode, scanning electron microscopy (SEM, JSM-7001FA) coupled with energy-dispersive X-ray spectroscopy (EDS) and atomic force microscopy (AFM, Bruker Nanoscope, Germany) in tapping mode were employed. The physicochemical properties of the GF and functionalized GF electrodes was investigated by micro-Raman spectroscopy using a 50 mW Nd: YAG (neo dymium-doped yttrium aluminium garnet) laser at 532 nm as excitation sources (RMP-300, JASCO Corporation, Japan). The analysis of the scattered beam was performed on a 250 mm focal length spectrometer along with an 1800 lines/mm diffraction grating and a high sensitivity CCD detector, while the frequency shifts were calibrated by an internal Si reference. X-ray photoelectron spectrophotometer (XPS) measurements (ULVAC PHI5000 VersaProbe) was performed to investigate the formation of oxygenated surface groups after functionalization. Different degrees of oxygenated surface groups present in as-prepared graphene biosensors can be evidenced by the Raman spectra and XPS analysis. These groups can provide essential sites for linking agents and subsequent attachment of specific antibodies and toxin molecules, i.e. MC-LR.

The electrochemical properties of the GF electrodes were first evaluated using cyclic voltammetry (CV) in a typical three-electrode cell (i.e. a working, an auxiliary and a reference electrodes) at ambient temperature on a potentiostat analyzer (Autolab PGSTAT128N plus an FRA32M module). The GF electrodes was characterized by CV in 2 mM potassium ferricyanide (K_3_[Fe(CN)_6_]) solution using 0.5 M KNO_3_ as the supporting electrolyte, with a Pt wire (1 mm diameter, 99.95%) as auxiliary electrode and a saturated Ag/AgCl electrode as the reference. Faradaic electrochemical impedance spectroscopy (EIS) analysis was carried out with an open-circuit potential from 0.1 to 1 × 10^4^ Hz with the amplitude of 0.01 V in the same three-electrode cell configuration but in a solution of 5.0 mM potassium ferricyanide (K_3_[Fe(CN)_6_])/potassium ferrocyanide (K_4_[Fe(CN)_6_]) solution using 0.5 M KNO_3_ as the supporting electrolyte.

### Preparation of GF biosensor and detection principle of MC-LR in water

To fabricate the GF biosensor, a pre-cut GF sheet (0.6 × 0.6 cm) was attached to a copper wire tip using conductive silver epoxy (M. G. Chemicals, Ontario, Canada) and dried for 24 h at room temperature. This was followed by electrochemical functionalization, where a potential of 1.2 V vs. Ag/AgCl was applied to GF biosensor tips for 1 min, in 1.0 M NaOH/0.5 M NaCl aqueous solutions. The functionalization potential (i.e. 1.2 V) was determined by choosing highest generated current on the surface of GF biosensor in linear sweep voltammetry (LSV) without observations of water hydrolysis (see Fig. S1). 0.5 M KNO_3_ and NaOH solutions with different concentration (0.25 to 1.0 M) was used as oxidizing solutions, which was similar to previous studies^34^.

Figure 1(b) shows the sequential steps of following bioconjugation. Zero-length cross linkers ethyl-3-(3-dimethylaminopropyl) carbodiimidehydrochloride (EDC) and *N*-hydroxysulfosuccinimide (sulfo-NHS) are commonly used to conjugate a range of biomolecules (i.e. enzymes, antibodies, peptides, DNA, fluorophores, etc.) to various nanomaterials’ surfaces without the need of prior modification^35^. Specifically in this work, the GF biosensors were first incubated in a solution of 5 mM sulfo-NHS and 2 mM EDC (ThermoFisher Scientific Inc.) in 0.1 M 4-morpholinoethanesulfonic acid (MES) (Acros Organics) buffer, which was followed by 4 h incubation in 500 μg/L MC-LR (Enzo Life Sciences) solution (5 mL) made with phosphate buffer saline (PBS) buffer (pH 7.4). Following that, 1% (v/v) ethanol amine was used to washed them thoroughly. As a result, amine group of MC-LR was conjugated to the oxygen containing functional groups (i.e. carboxyl group) on the surface of the electrochemically functionalized GF electrode through a stable covalent link^36^. The sulfo-NHS was added to increase the stability of the formed amine-reactive intermediate by converting it to an amine-reactive sulfo-NHS ester and thus avoid its rapid hydrolysis in aqueous solutions. Next, MC-LR solutions of a range of concentrations from 0.005 to 10 μg/L were added into a fixed concentration of monoclonal antibodies (i.e. 2.2 μg/mL) (against ADDA, AD4G2, mouse IgG1 from Enzo Life Sciences) and incubated for 30 min to prepare a series of incubation standard solutions (i.e. 0.005, 0.05, 0.5, 2.5 and 10 μg/L) using PBS buffer. The GF biosensors saturated with 500 μg/L MC-LR solution in the previous step were then dipped into these incubation standard solutions, where remaining free antibodies in the solutions would bind to the MC-LR on the GF biosensors. Finally, these GF biosensors were rinsed with PBS solution and immersed in a prepared 5.0 mM K_3_[Fe(CN)_6_] solution, where charge transfer resistance value of GF biosensors was measured using Faradaic EIS and linearly fitted to the MC-LR concentration of different incubation standard solutions. EIS measurement of each MC-LR concentration was repeated three times with three different fabricated GF biosensors.

### Environmental water sample analysis and validation procedure

To simulate the water contamination event by MC-LR toxins, four environmental water samples were selected, i.e. Tokyo metropolitan tap water, Sanshiro pond water (Tokyo, Japan), Shinobazu pond water (Tokyo, Japan) and Inba lake water (Chiba prefecture, Japan). All the environmental water samples used in this study were pre-filtered using 0.2 μm pore size PTFE disk filter (Advantec 25HP020AN). Since no trace of MC-LR was detected, a fixed amount of MC-LR (i.e. 0.1 μg/L) were spiked into all environmental water samples to simulate the water contamination.

High resolution Fourier transform mass spectrometry (FT-MS, Themo Fisher Scientific Exactive 1.1 Orbitrap Mass Spectrometer) was used to validate the electrochemical sensing results by developed GF biosensors. It was operated in negative mode and deionized water was used as mobile phase. Fingerprint peak of MC-LR is expected to show at m/z of 993.5 in negative mode with an m/z detection range of between 1 and 1000. Environmental water samples for FT-MS analysis was prepared via solid phase extraction (SPE) method (Agilent Life Sciences 12255002 Bond Elut-PPL, 1 gm 6 mL), which was followed step-by-step as in reference^37^.

## Results and Discussion

### Characterization of as-prepared GF electrode

**Figure 2:**
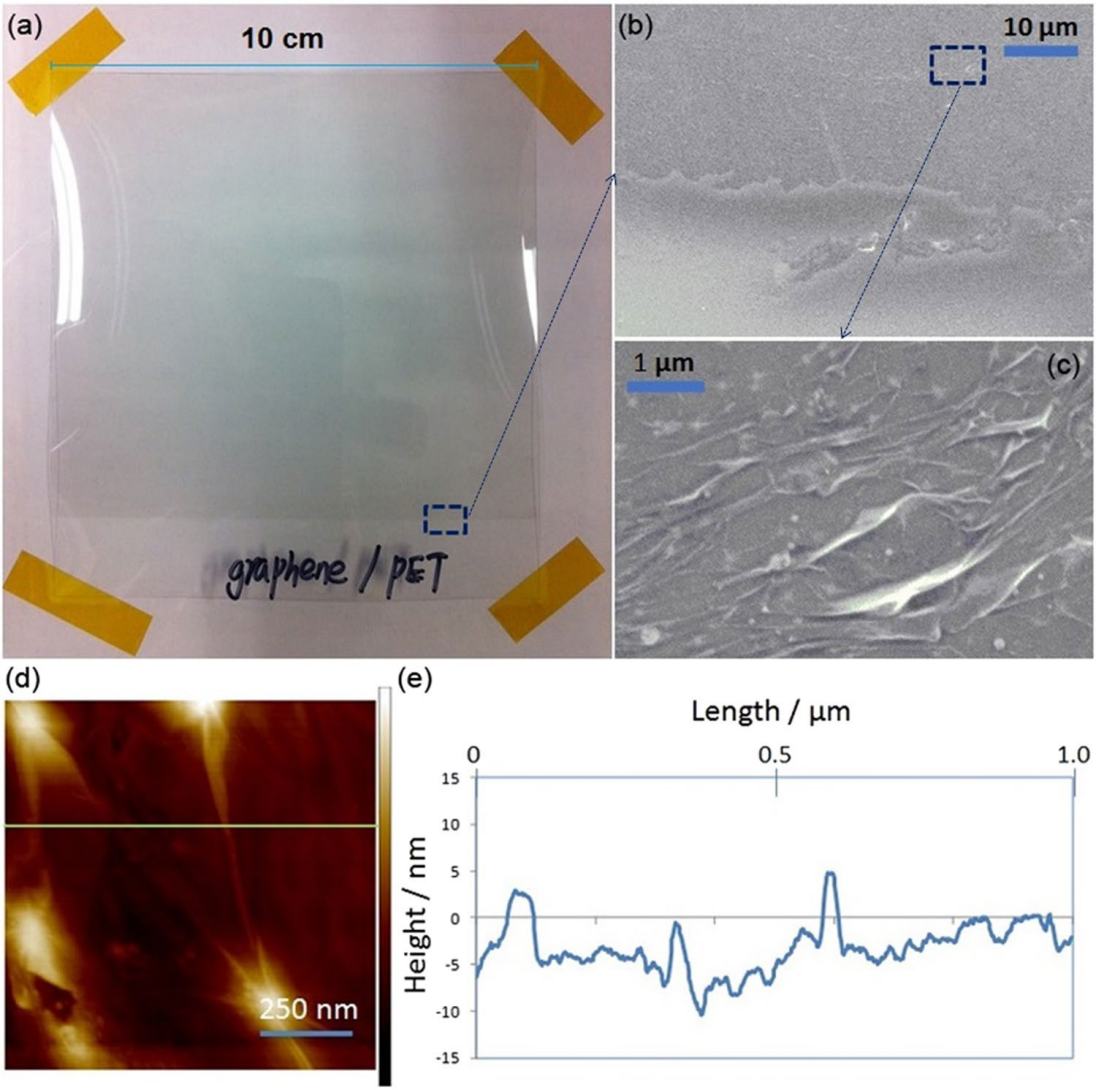
(**a**) A photographic image of graphene/PET composite; (**b**) SEM of graphene/PET composite; (**c**) enlarged SEM image of selected area on graphene/PET composite; (**d**) AFM images of graphene/PET composite and (**e**) height profile of graphene film coating along the green line in (**d**).

Figure 2(a) shows a macroscopic photo of as-prepared graphene/PET composite, where a horizontal straight line is visible and marks the partition between naked PET section (i.e. transparent below) and the one with graphene film coating (i.e. slightly greyish above). As seen in Fig. 2(b), the corresponding SEM image of the marked area in Fig. 2(a) shows the partition line in higher magnification. Further enlarged image in Fig. 2(c) demonstrates the details surface morphology of graphene film, which featured many creases and wrinkles. This could potentially contribute to the increase of surface area, which is essential for effective biomolecules adsorption. AFM image in Fig. 2(d) further confirmed the surface roughness of graphene film coating. Figure 2(e) further illustrates the height profile of graphene film coating, where it can be seen that deposited graphene film was quite rough and between 5 to 10 nm thick on average. Estimated thickness of single layer graphene was reported to be between 0.4 and 1.7 nm in literature^38^ and thus graphene film on PET substrates varied roughly between five to nine layers graphene stacking.

**Figure 3:**
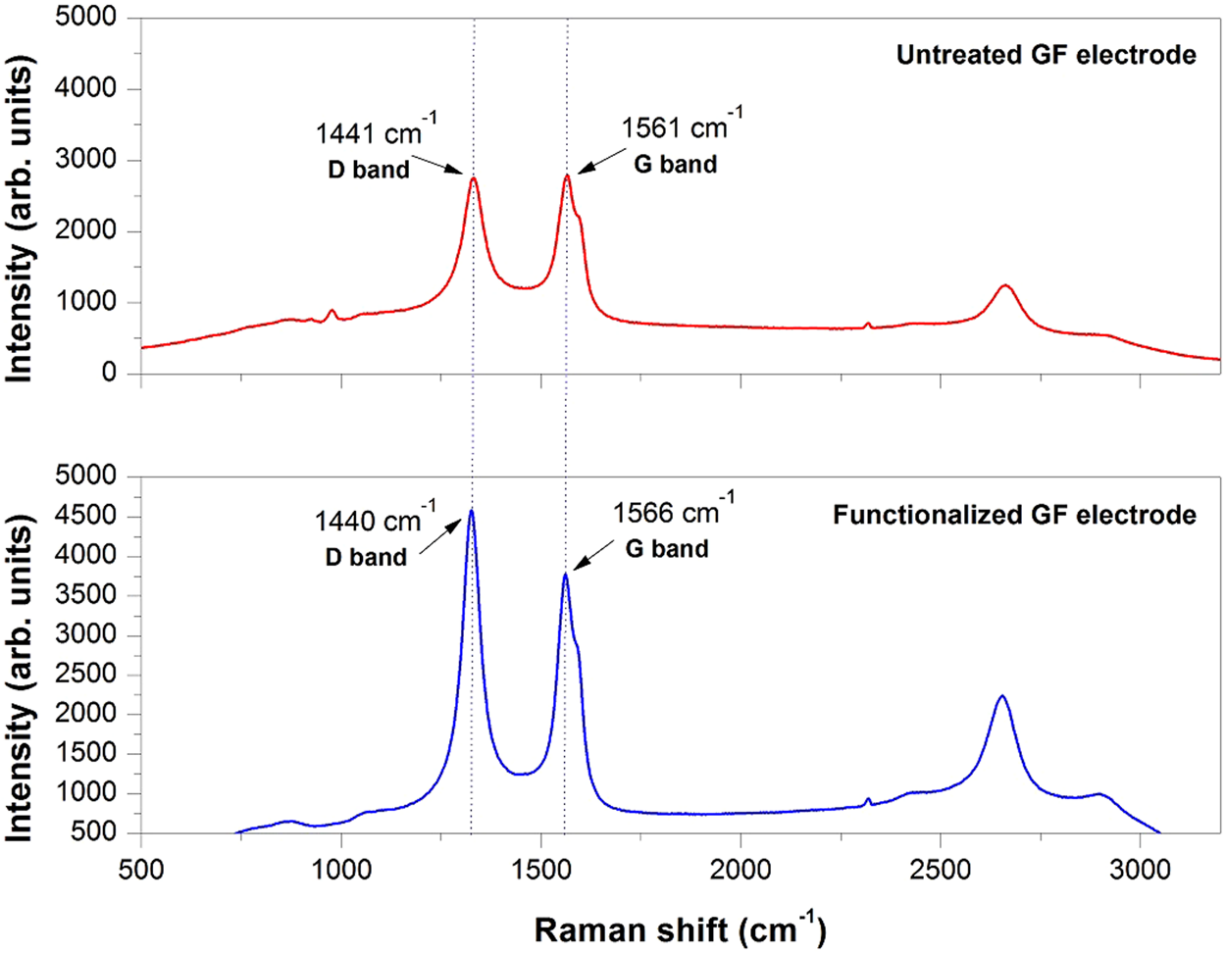
Typical Raman spectra of graphene film and functionalized graphene film electrode in this work.

Figure 3 shows the Raman spectra of untreated GF and functionalized GF electrode, where G bands at around 1480–1580 cm^−1^ correspond to in-plane vibration of sp^2^ bonded carbon structure and D bands at about 1320–1450 cm^−1^ mainly reflect defects and disorders in the graphitic structure. The intensity ratio of D bands and G bands is usually used to evaluate the graphitization degree of carbon. The I_D_/I_G_ of functionalized GF electrode was calculated to be 1.215, implying the domination of disordered or amorphous structure. In comparison, the I_D_/I_G_ of untreated GF electrode decreased to 0.98, indicating increased graphitization degree of graphene. This is more likely caused by electrochemical oxidation and introduction of surface oxygen-containing functional groups.

**Figure 4:**
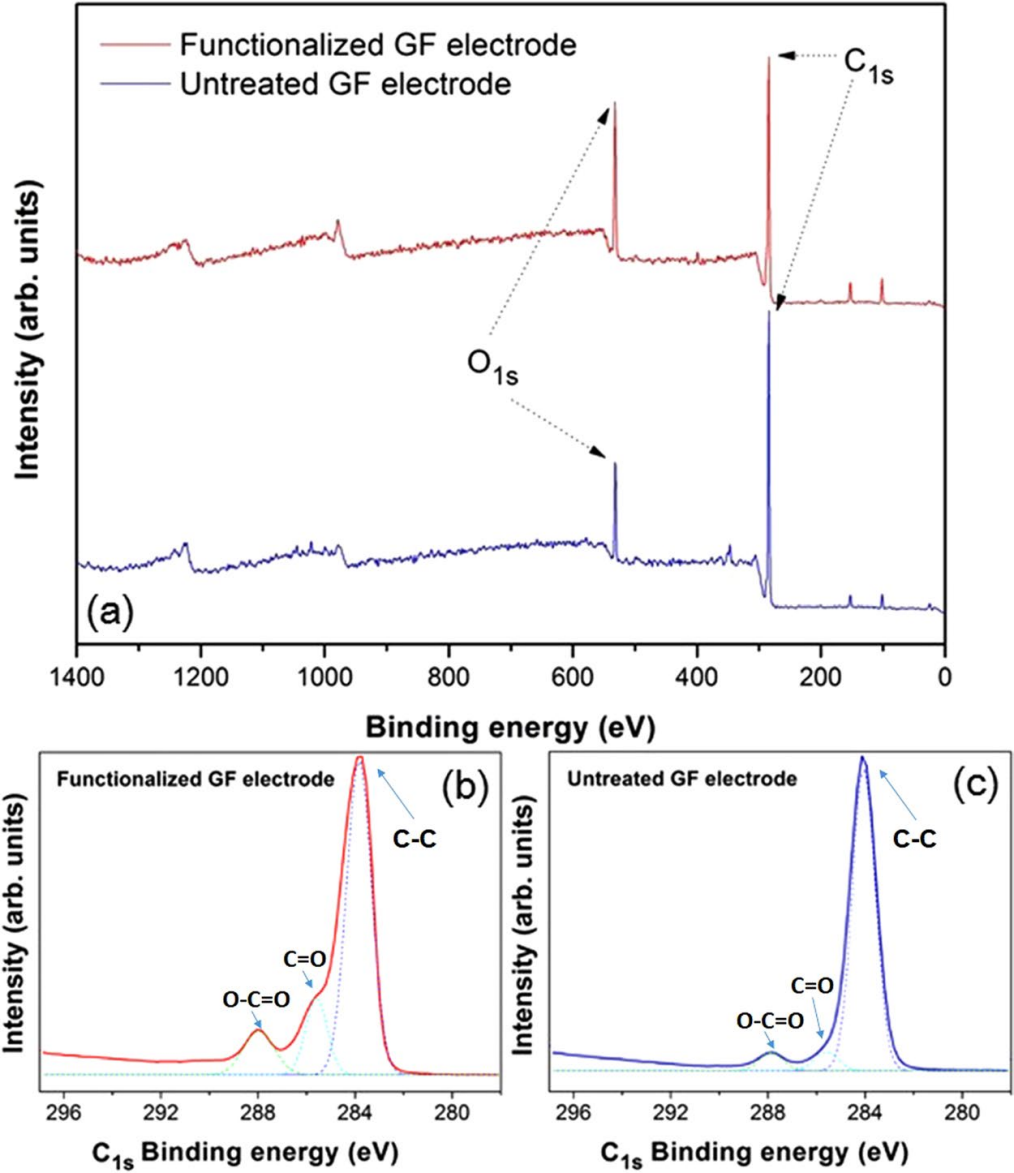
XPS spectra of full survey (**a**) and deconvoluted C_1s_ (**b, c**) binding energy region for graphene film electrodes before and after surface electrochemical oxidation, where solid and dash lines represent the experimental data and the single-peak gaussian profile of each designated binding energy region, respectively.

Figure 4 depicts the XPS survey spectra on the surface of developed graphene film biosensor before and after surface electrochemical oxidation, where peak areas around 284 eV and 532 eV are typical C_1s_ and O_1s_ binding energy region, respectively. The deconvoluted C_1s_ XPS signals of GF biosensors in Fig. 4(b,c) displays peak components at binding energies of ~284, ~285.8 eV and ~287.9, assigned to graphitic carbon (C-C), carbonyl (C=O) and carboxyl (O-C=O) groups^39^. After electrochemical functionalization, relative intensity of carbonyl (C=O) and carboxyl (O-C=O) peak components were observed to increase drastically. In addition, the ratio of oxygen to carbon (O/C) atoms ratio on the surface of untreated graphene film electrode was 0.15 while that on the surface of oxidized graphene film electrode increased to 0.3, indicating that electrochemical oxidation successfully introduced more oxygenated content (i.e. carboxyl functional group) into the graphene structure. On other hand, higher oxygen to carbon atom ratio after surface oxidation means lower graphitization degree of carbon, which was also in good agreement with Raman spectra results of the same samples.

### Electrochemical properties and MC-LR detection using developed GF biosensors

**Figure 5:**
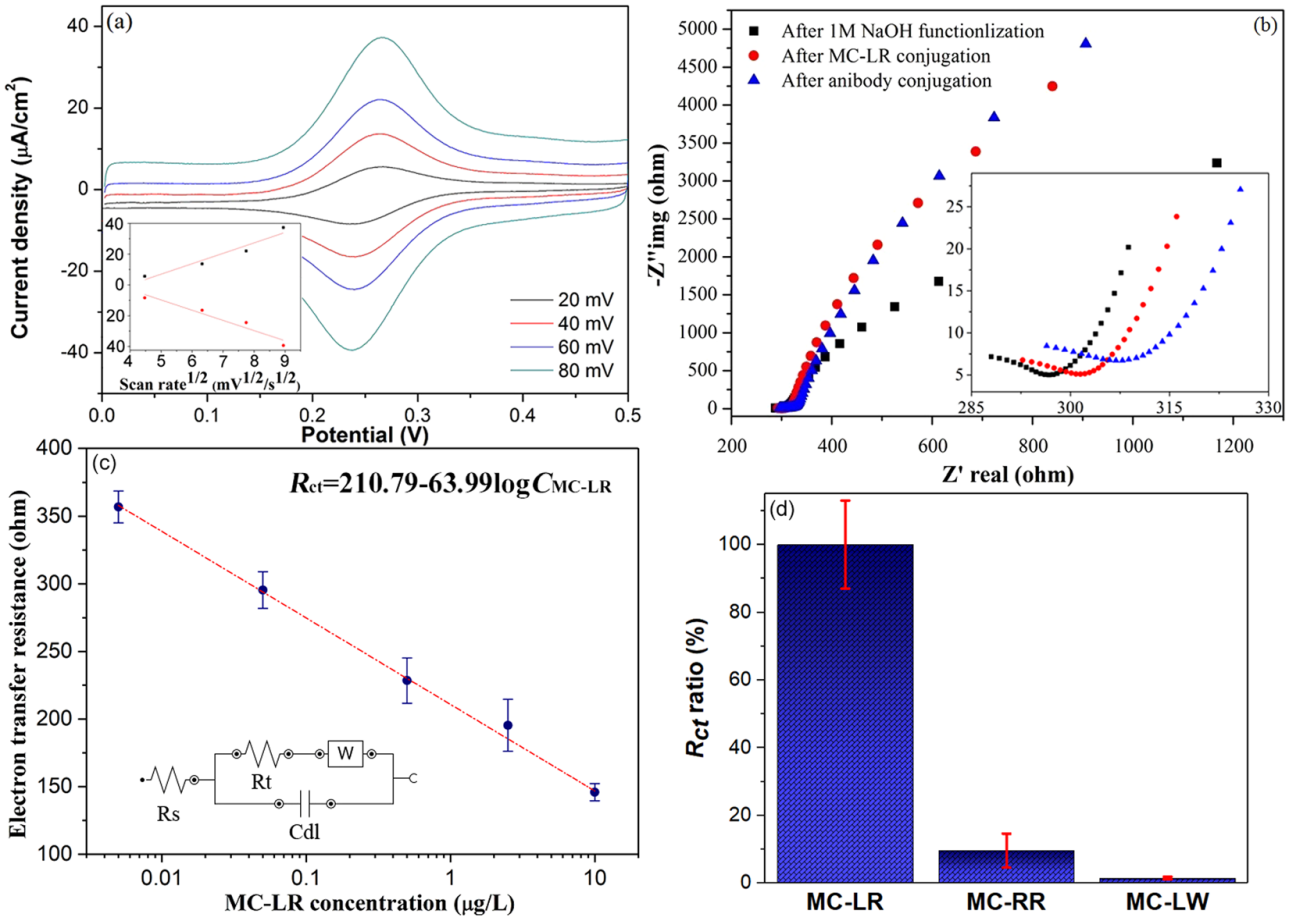
(**a**) Typical CV plots of developed GF biosensor in 2 mM K_3_[Fe(CN)_6_]/0.5 M KNO_3_ solution at different scan rates, where the inset is the anodic peak (top) and catholic peak current density (bottom) in µA/cm^2^ plotted against scan rate^1/2^ in mV^1/2^/s^1/2^; (**b**) Nyquist plots for EIS measurements of GF biosensor after their surface electrochemical oxidation (i.e. functionalization in 1 M NaOH solution), MC-LR, and antibody conjugation, where the inset is the enlarged section of beginning; (**c**) charge transfer resistance changes of GF biosensor as a function of MC-LR concentrations between 0.005 and 10 μg/L, where the inset is the representation of Randles circuit (error bars: SD, n = 3); (**d**) cross-reactivity test of as-prepared GF biosensor on MC-RR and MC-LW against MC-LR in terms of *R*_*ct*_ ratios, where all of their concentration was 1 µg/L (error bars: SD, n = 3).

Typical CV behaviour of developed GF biosensor after surface electrochemical treatment was recorded as functions of different scan rates. In Fig. 5(a), well-defined reversible reduction (at ~0.23 V)/oxidation (at ~0.27 V) peak typically corresponding to the Fe^III^/Fe^II^ redox couple indicated efficient charge transfer between the GF biosensor and the electro-active species in solution (2 mM K_3_[Fe(CN)_6_]/0.5 M KNO_3_) and thus their suitability as sensing materials. In the inset of Fig. 5(a), a linear relationship between both cathodic (at ~0.23 V) and anodic peak (at ~0.27 V) currents from the GF biosensor and the square root of scan rate indicated that the Fe^III^/Fe^II^ redox reaction occurred on GF biosensor was a reversible and diffusion-controlled electrochemical process^40^.

Nyquist plots (i.e. Z_imag_ vs. Z_real_ spectra) for EIS measurements of GF biosensors was carried out and exhibited in Fig. 5(b) as three separate plots after their surface electrochemical oxidation, MC-LR and antibody conjugation, respectively. For Faradaic electrochemical impedance biosensors in the presence of a redox couple (i.e. Fe(CN)_6_^3−^/Fe(CN)_6_^4−^), Nyquist plots typically consist of a semicircle at higher frequencies that corresponds to the electron-transfer-limited process followed by a linear part at lower frequencies due to the diffusion-limited processes^41,42^. The anchoring of target biomolecules onto the electrode could reduce its available surface area, form an insulating layer and thus inhibit the interfacial charge transfer process. On the other hand, the presence of negative charges on conjugated biomolecules (i.e. MC-LR/antibody) could induce extra electrostatic repulsion upon the approach of redox active ion pair (i.e. Fe(CN)_6_^3−^/Fe(CN)_6_^4−^) to the surface of GF biosensor, which could lead to further resistance increase of biosensor electron-transfer. As a consequence, this gradual increase in the electron-transfer resistance of the GF biosensor after the conjugation with MC-LR and antibodies was clearly evidenced by an increase in the semicircle diameter in the inset of Fig. 5(b). These observations are consistent with previous studies, which reported the similar increase in the electron-transfer resistance induced by the relatively larger biomolecule complexes formation on the surface of sensor electrode^43–45^.

The GF biosensor response (i.e. the electron-transfer resistance) as a function of different MC-LR incubation solution concentrations was plotted in Fig. 5(c). A Randles model equivalent circuit in the inset of Fig. 5(c) were used during the EIS measurements to interpret the behaviour of the GF biosensors, which consisted the solution resistance (*R*_s_) in series with a parallel arrangement of the double layer capacitance (*C*_dl_) and the electron-transfer resistance (*R*_ct_) in series with Warbur impedance (*Z*_w_). The determined values of the *R*_ct_ were then plotted as a function of the MC-LR concentration using a logarithmic scale. As described in the MC-LR detection procedure of the experimental section, since the amount of antibodies was fixed and MC-LR concentration was varied in the incubation solutions, the remaining amount of unbound antibodies in the solution after the MC-LR conjugation should be thus inversely proportional to the concentration of MC-LR. Therefore, after the conjugation of remaining unbound antibodies with the GF biosensor, the electron-transfer resistance of GF biosensors increased with the decrease of the MC-LR concentration. A great linear sensing response (*R*^2^ = 0.99) of the electron-transfer resistance (*R*_ct_) change over a wide MC-LR concentration (*C*_MC-LR_) range between 0.005 and 10 μg/L was thus achieved, which allowed the toxin detection well below the WHO provisional guideline limit of 1 μg/L for MC-LR in drinking water. The limit of detection (LOD) was calculated to be 0.0023 μg/L (commonly defined as LOD = 3 × standard deviation of blank/slope of calibration plot^46^). Intra-assay reproducibility of fabricated GF biosensors was assessed by replicative EIS measurement of each one of five MC-LR concentrations (i.e. 0.005 to 10 µg/L) using three different GF biosensors, of which the relative standard deviations (RSD) were 4.5%, 7.3%, 13%, 10.8% and 5.2%, respectively. These results have demonstrated developed GF biosensor as a very effective tool for monitoring the variation of MC-LR concentration in water in terms of good reproducibility.

For cross-reactivity test of as-prepared GF biosensors, most common MC variants present in freshwater (i.e. 1 µg/L MC-RR and MC-LW, Enzo Life Sciences) were also incubated with GF biosensors, and then conjugated with MC-LR antibody. The follow-up EIS results was compared with MC-LR incubation of same concentration using same procedure. Figure 5(d) showing that EIS response (i.e. *R*_ct_) after antibody conjugation produced by the same concentration of MC-RR was less than 10% of that produced by MC-LR, while the interference of MC-LW was almost negligible due to much larger difference in molecular structure from MC-LR. These results were consistent with previous report^47^ and has demonstrated the high selectivity of monoclonal antibody, on which the current electrochemical GF biosensor were based.

### Environmental water sample analysis and GF biosensor validation

Performance validation for developed GF biosensor detection of MC-LR was carried out by using four different environmental water samples, of which the major water quality indexes are summarized in Table S1. Except for the tap water, other three environmental water samples have similar TOC level. Other characteristics of four environmental water samples (i.e. chemical composition) were further analyzed using three-dimensional fluorescence excitation–emission matrix (FEEM) spectra, which is shown in Fig. S2. Due to the low TOC value, no significant FEEM intensity area is observed in Tokyo tap water sample. Main dissolved organic compounds in Inba Lake samples are identified to be substances of fulvic acid nature, whereas Shinobazu and Sanshiro pond samples feature the presence of both fulvic acid and aromatic proteins with humic alike substances to a much less extent. GF biosensor detection results of MC-LR in four different environmental water samples are listed in Table S1, where it can be seen that potential interference (i.e. metal ions and dissolved organic compounds) has insignificant effects on the detection results. In addition, electrochemical sensing results by developed GF biosensors was also compared with more established analytical technique for MC-LR detection in literature, i.e. high-resolution Fourier Transform Mass Spectrometry (FT-MS). As seen in Figure S3(a), FT-MS could detect MC-LR well in μg per litre level via the peak intensity (Ip) at m/z 993.5, which makes it competent as baseline comparison with GF biosensor detection results. In addition, there was also an excellent linear response (R^2^ = 0.99) achieved between the peak intensity (Ip) at m/z 993.5 and the same range of MC-LR concentrations (C_*MC-LR*_) between 0.005 and 10 μg/L in Figure S3(b). Consequently, FT-MS detection results of the same environmental water samples are very similar to that using GF biosensors (Table 1). Hence, developed GF biosensor technique in this work has shown its potential as a simple and efficient alternative screening method for fast, sensitive and quantitative assessment of MC-LR detection in the event of local water contamination, especially *in-situ*. Due to the inevitable slow deterioration of surface-attached biomolecules during the storage, the stability of GF biosensor was also tested by storing the biosensor at 4 °C in PBS solutions for an extended period of time. After one- and two-weeks storage, 92.5% and 83.6% of initial EIS responses level were obtained using the same tap water sample, respectively. Lastly, MC-LR detection performance of as-prepared GF biosensor was also demonstrated to be very comparable to most of recent literature using nanomaterial biosensor based on different electrochemical detection principles (see Table 2), however, fabrication technique of sensor tips reported in work is more scalable producing biosensor tips with easily tunable size.

**Table 1:** Validation results of MC-LR detection using GF electrochemical biosensor and FT-MS method.

	GF biosensor value (μg /L)	Recovery (%)	FT-MS value (μg /L)	Recovery (%)
Tokyo tap water	0.0982 ± 0.0011	98.2	0.0993 ± 0.0009	99.3
Inba lake water	0.0963 ± 0.0023	96.3	0.0987 ± 0.0013	98.7
Sanshiro pond water	0.0935 ± 0.0031	93.5	0.0955 ± 0.0029	95.5
Shinobazu pond water	0.0941 ± 0.0013	94.1	0.0963 ± 0.0021	96.3

**Table 2:** MC-LR detection performance using most recent immuno-electrochemical biosensors in literature.

LOD (ng/L)	Detection range (µg/L)	Recovery (%)	Detection techniques	References
—	0.05 to 100	92.7	Electrochemical impedance spectroscopy	^33^
—	0.05 to 100	—	Electrochemical impedance spectroscopy	^34^
4	0.01 to 100	94.1–98.1	Electrochemical impedance spectroscopy	^46^
4	0.005 to 50	91.6–110.7	Cyclic voltammetry	^48^
1.68	0.0025 to 5	98–99.2	Differential pulse voltammetry	^49^
2.3	0.005 to 10	93.5–98.2	Electrochemical impedance spectroscopy	Current study

## Conclusions

A fit-for-purpose biosensor for MC-LR detection as alternatives to the time-consuming, expensive, non-portable and often skills-demanding conventional methods of analysis involved in water quality assessment has been developed using GF/PET composite grown by a modified CVD method. A three-step linking procedure that enabled the immobilization of MC-LR onto the GF electrodes and conjugation of monoclonal antibodies specific to MC-LR in the incubation solutions has been employed to provide the required specificity for detecting MC-LR toxin. The increase of electron transfer resistance upon bioconjugation of MC-LR and antibodies on GF electrodes was used to detect the change of MC-LR concentration. A great linear sensing response (*R*^2^ = 0.99) of EIS change was established over a wide MC-LR concentration range of 0.005 to 10 μg/L with good reproducibility. Environmental water samples from different local sources, i.e. Tokyo metropolitan tap, Sanshiro pond (Tokyo, Japan), Shinobazu pond (Tokyo, Japan) and Inba lake water (Chiba prefecture, Japan), has been used to validate the sensing results of developed GF biosensors and investigate potential interfering effects of factors, such as metal ions and dissolved organic compounds. With insignificant interference effects on the detection results and high MC-LR detection recovery, developed GF biosensor in this work has provided one more step further towards quick *in-situ* detection of MC-LR in the contamination point of water source with very low detection limits (i.e. 2.3 ng/L). Developed graphene film composite biosensors offers a promising possibility of large-scale manufacture of sensor tips due to their macroscopic free-standing nature, scalable fabrication route and easily tunable size.

## Electronic supplementary material

Supporting Information
