# Supplementary material for: Fabrication of graphene film composite electrochemical biosensor as a pre-screening algal toxin detection tool in the event of water contamination
Source: Sci Rep. 2018 Jul 16;8:10686. doi: 10.1038/s41598-018-28959-w (PMC6048102; doi:10.1038/s41598-018-28959-w)
Supplement: Supplementary file 1 — Supporting Information [file 41598_2018_28959_MOESM1_ESM.docx]

**Supporting Information**

**Fabrication of graphene film composite electrochemical biosensor as a pre-screening algal toxin detection tool in the event of water contamination**

Wei Zhang^1,3,4*^, Baoping Jia^2^ and Hiroaki Furumai^1^

1. *Research Centre for Water Environment Technology, Department of Urban Engineering, The University of Tokyo, Tokyo 113-0033, Japan.*
2. *School of Materials Science and Engineering, Changzhou University, Changzhou, Jiangsu 213164, China*
3. *Systems and Process Engineering Centre, College of Engineering, Swansea University, Swansea University, Bay Campus, Swansea SA1 8EN, UK.*
4. *School of Natural and Built Environments, University of South Australia, Mawson Lakes, South Australia 5095, Australia.*

^*^Corresponding author:

Tel.: +81-03-58416248

Email: [zhangwei@env.t.u-tokyo.ac.jp](mailto:zhangwei@env.t.u-tokyo.ac.jp) (W. Zhang)


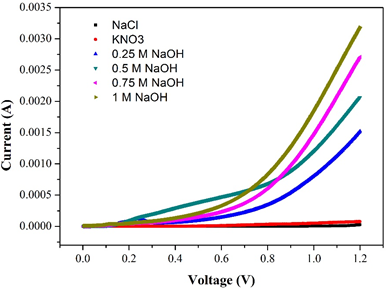


Figure S1 Current vs voltage response of as-prepared GF biosensor tips in different oxidizing solutions.

Conductivities and total dissolved solids (TDS) of water samples were measured using a hand-held meter (Lutron CD-4302). Major metal ions concentrations in water samples such as Fe^3+^, Mg^2+^, and Cu^2+^, etc. were investigated using an inductively coupled plasma mass spectrometry (ICP-MS, Agilent 7500 Series, USA). All glassware for the preparation of samples were pre-washed by 10% nitric acids solution. Total organic carbon (TOC) values of water samples were obtained using a TOC analyzer (Shimadzu, Japan).

**Table S1** Environmental water samples qualities used in this work

| **Water quality parameters** | **Tokyo tap** | **Inba Lake** | **Sanshiro pond** | **Shinobazu pond** |
| --- | --- | --- | --- | --- |
| TOC (mg/L) | 0.53 | 7.91 | 8.99 | 8.68 |
| UV254 (cm^-1^) | 0.006 | 0.048 | 0.059 | 0.039 |
| pH | 7.9 | 8.6 | 7.2 | 8.5 |
| Conductivity (μS/cm) | 256 | 307 | 184 | 265 |
| Total dissolved solids (mg/L) | 130 | 158 | 118 | 170 |
| Sodium (mg/L) | 5.64 | 2.62 | 3.02 | 7.46 |
| Magnesium (mg/L) | 6.32 | 6.76 | 4.9 | 14.67 |
| Aluminium (µg/L) | 0.47 | 0.25 | 0.35 | 0.29 |
| Potassium (mg/L) | 3.56 | 2.95 | 0.58 | 0.02 |
| Calcium (mg/L) | 2.93 | 2.82 | 2.87 | 2.29 |
| Manganese (µg/L) | 0.49 | 1.85 | 14.32 | 0.68 |
| Iron (µg/L) | 15.37 | 0.86 | 174.2 | 8.26 |
| Copper (µg/L) | 4.59 | 7.99 | 1.19 | 1.57 |

Fluorescence excitation–emission matrix (FEEM) analysis was utilised to identify the chemical composition of dissolved organic matter because of its ability to distinguish among certain classes of organic matter in environmental water samples [^[[1]](#endnote-1)^]. The fluorescence intensity of the feed and filtrate was measured at excitation wavelengths between 200 and 450 nm, and emission wavelengths between 230 and 550 nm in 5 nm increments, with a three-dimensional fluorescence spectrophotometer (Agilent Cary Eclipse, USA). The excitation and emission slits were maintained at 10 nm, and the scanning speed was set at 1200 nm/min for this study. The spectrum of deionized water was recorded as the blank. Fluorescence signals detected in specific areas are related to specific fractions of dissolved organic carbon (DOC), based on previous studies [^[[2]](#endnote-2)^,^[[3]](#endnote-3)^].

There are five key fluorescence region commonly observed in freshwater samples: regions I (Ex/Em = 200–250/280–330) and II (Ex/Em = 200–250/330–380) are related to simple aromatic proteins like tyrosine and tryptophan (P1 and P2); region III (Ex/Em = 200–260/380–500) is attributed to fulvic acid-like material; region IV (Ex/Em = 250–380/280–380) is closely related to soluble microbial product (SMP) [^[[4]](#endnote-4)^]; region V (Ex/Em = 250–380/380–500) is associated with humic-like substances derived from the breakdown of plant material [^[[5]](#endnote-5)^]. Due to the low TOC value, no intensity area in tap water sample is observed compared with other three environmental samples. Absence of any noticeable peaks in region IV and V suggested no presence of any microbial product and humic alike substances in Inba Lake samples, whereas dominant intensity areas are located in region III of fulvic acid nature. In cases of Shinobazu and Sanshiro pond samples, the main intensity peaks are identified to be substances of fulvic acid nature at region III and aromatic proteins at region I and II, and to a much less extent humic alike substances at region V.


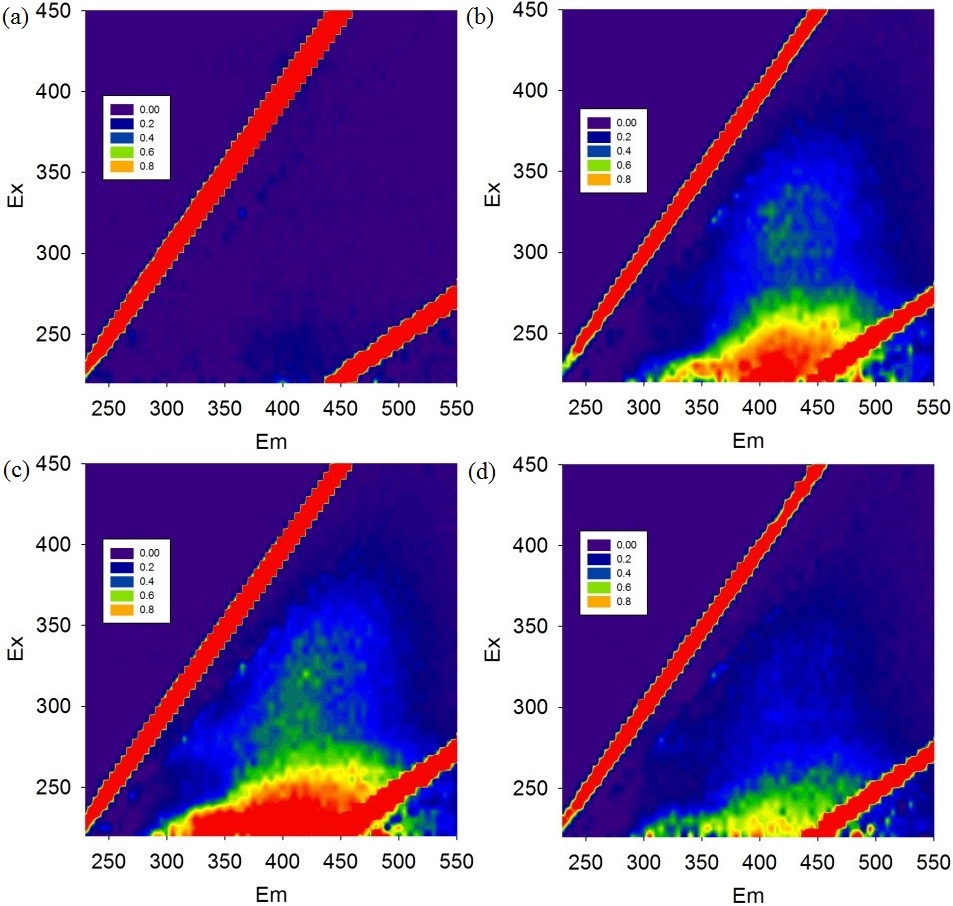


Figure S2 FEEM spectra of four environmental water samples: (a) Tokyo tap water, (b) Shinobazu pond water, (c) Sanshiro pond water and (d) Inba Lake water.


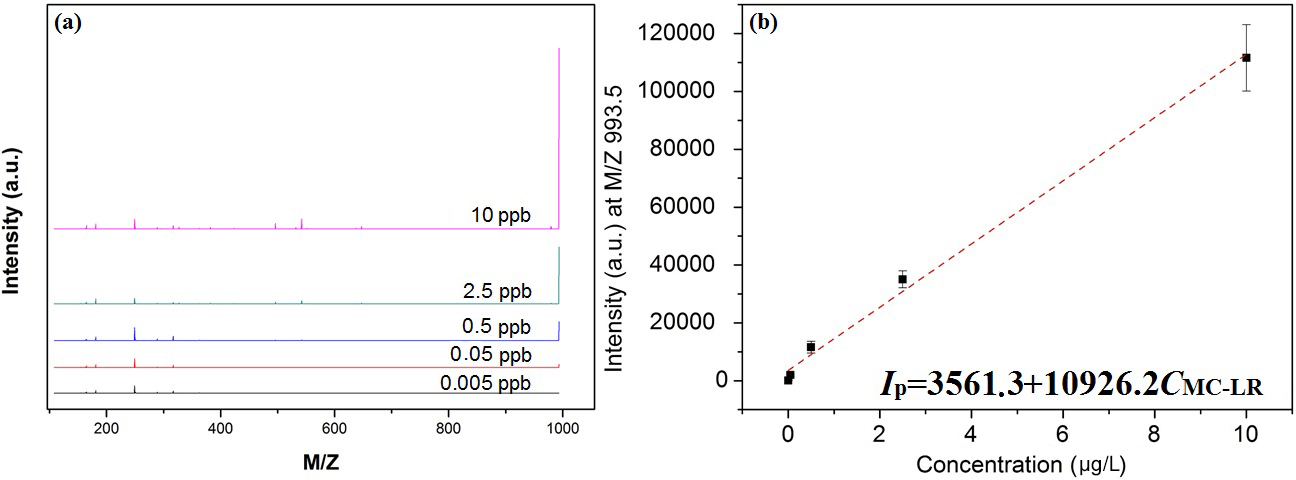


Figure S3 (a) FT-MS detection spectra and (b) the linear response of peak intensity (*I*_p_) at m/z 993.5 as function of MC-LR concentrations (*C*_MC-LR_) between 0.005 and 10 μg/L (error bars: SD, n=3).

# References

1. [] Saadi, I., Borisover, M., Armon, R. & Laor, Y. Monitoring of effluent DOM biodegradation using fluorescence, UV and DOC measurements, *Chemosphere* **63**, 530–539 (2006). [↑](#endnote-ref-1)
2. [] Zhang, W., Zhang, Y., Fan, R., Lewis, R. A facile TiO_2_/PVDF composite membrane synthesis and their application in water purification, *Journal of Nanoparticle Research*, **18**, 31 (2016). [↑](#endnote-ref-2)
3. [] Chun, Y., [Zaviska, F.](https://www.scopus.com/authid/detail.uri?authorId=25936995700&amp;eid=2-s2.0-84910145854), [Cornelissen, E.](https://www.scopus.com/authid/detail.uri?authorId=15131229600&amp;eid=2-s2.0-84910145854), [Zou, L.](https://www.scopus.com/authid/detail.uri?authorId=55994643200&amp;eid=2-s2.0-84910145854) A case study of fouling development and flux reversibility of treating actual lake water by forward osmosis process, *Desalination*, 357, 55-64 (2015). [↑](#endnote-ref-3)
4. [] K. C. Khulbe, C. Y. Feng, T. Matsuura, Membrane characterization. Membrane processes. United Nations Educational, Scientific and Cultural Organization, 2010, pp 131–172. [↑](#endnote-ref-4)
5. [] J. I. Calvo, A. Hernández, P. Prádanos, L. Martınez, W. R. Bowen, Pore size distributions in microporous membranes ii. Bulk characterization of track-etched filters by air porometry and mercury porosimetry, *Journal Colloid. Interface Science* **176**, 467–478 (1995). [↑](#endnote-ref-5)
